# Men are the main COVID-19 transmitters: behavior or biology?

**DOI:** 10.1007/s44192-022-00004-3

**Published:** 2022-01-24

**Authors:** Monize V. R. Silva, Mateus V. de Castro, Maria Rita Passos-Bueno, Paulo A. Otto, Michel S. Naslavsky, Mayana Zatz

**Affiliations:** 1 Human Genome and Stem Cell Research Center (HUG-CELL), Biosciences Institute, University of Sao Paulo, Sao Paulo, SP Brazil; 2 Department of Genetics and Evolutionary Biology, Biosciences Institute, University of Sao Paulo, Sao Paulo, SP Brazil

**Keywords:** COVID-19, Household transmission, Behavior, Adult couples, SARS-CoV-2

## Abstract

**Background:**

COVID-19 has affected millions of people worldwide. Clinical manifestations range from severe cases with lethal outcome to mild or asymptomatic cases. Although the proportion of infected individuals does not differ between sexes, men are more susceptible to severe COVID-19, with a higher risk of death than women. Also, men are pointed out as more lax regarding protective measures, mask wearing and vaccination. Thus, we questioned whether sex-bias may be explained by biological pathways and/or behavioral aspects or both.

**Methods:**

Between July 2020 and July 2021, we performed an epidemiological survey including 1744 unvaccinated adult Brazilian couples, with there was at least one infected symptomatic member, who were living together during the COVID-19 infection without protective measures. Presence or absence of infection was confirmed by RT-PCR and/or serology results. Couples were divided into two groups: (1) both partners were infected (concordant couples) and (2) one partner was infected and the spouse remained asymptomatic despite the close contact with the COVID-19 symptomatic partner (discordant couples). Statistical analysis of the collected data was performed aiming to verify a differential transmission potential between genders in couples keeping contact without protective measures.

**Results:**

The combination of our collected data showed that the man is the first (or the only) affected member in most cases when compared to women and that this difference may be explained by biological and behavioral factors.

**Conclusions:**

The present study confirmed the existence of gender differences not only for susceptibility to infection and resistance to COVID-19 but also in its transmission rate.

## Highlights

- There are sex differences in COVID-19 susceptibility and transmission between couples highly exposed to SARS-Cov-2 without protective measures;
- Men are more efficient virus transmitters than women;
- Sex-bias in COVID-19 transmission can be explained by differences in viral load in saliva, immune response and also behavioral protective differences between genders.

## Introduction

Since the first reported case of COVID-19 in December 2019, some 200 million individuals have been infected by the novel coronavirus. Global epidemiological data revealed that age increases the risk of dying from COVID-19 because of the significant number of older adults with comorbidities [1–4]. Moreover, although there are no gender differences in the proportion of people infected by SARS-CoV-2, men are more susceptible to develop severe COVID-19 [5, 6].

COVID-19 pandemic caused a significant number of deaths worldwide, but the prevalence of comorbidities [7, 8], lower education levels, socioeconomic inequalities as the healthcare system inexperience to deal with the pandemic [9, 10] contributed to the increase of COVID-19 cases and deaths in low- and middle-income countries (LMICs) [11–13]. Most of these countries still show inadequate vaccination programs, lack of an accurate and rapid diagnosis as well as poor viral surveillance [10, 14–16], contributing for the emergence of COVID-19 waves and new SARS-CoV-2 variants [17, 18].

Independently of age, men are more likely to have complications by COVID-19 than women and if hospitalization is required, males are more at risk of death than females [19]. Interestingly, comparable gender differences also occur for other viral infections [20]. Furthermore, some behavioral aspects such as COVID-19 prevention and control measures vary between genders. A survey conducted in March–April 2020 indicated that men are more reluctant than women to wear protective masks and respect social distancing [21].

A recent survey conducted with almost 2000 American adults showed that the importance of masks for protection differ considerably between genders and males are more likely to consider masks as an infringement on their independence and freedom [22]. Another survey with 2500 Americans showed that males are less inclined to wearing masks than females, although both genders consider masks shameful [23]. Other protective practices as handwashing and sanitizer use had been previously reported as more common among females [24] and this tendency is the same for COVID-19 pandemic [25].

Interestingly, preliminary surveys to evaluate sex-differences for COVID-19 vaccination intention showed that females intended less to be vaccinated, probably due to the fear as to side effects or even the desire to “not the first to be vaccinated” [26–29]. Nonetheless, updated data compiled by The COVID-19 Sex-Disaggregated Data Tracker initiative by Global Health 5050 [30] has shown that more females are being vaccinated than males in many countries, including Brazil and U.S., as also shown by local media [31–33].

Considering the mental health impact due the pandemic, it was also observed that females experienced more depression symptoms after lockdown while males experienced more anxiety symptoms [34], but after a few weeks the gender-differences disappeared and both groups experienced resilience feelings [35]. The mental impact of the pandemic in LMICs varied from immediate distress and discrimination experienced mainly by healthcare workers to long-term side effects such as higher prevalence of depression and other mood disorders as a result of social isolation, job insecurity, unemployment and economic distress [36].

It has been suggested that some individuals (named “superspreaders”) are able to transmit the virus to a great number of persons [37]. However, it is not known if this could be explained by behavior (men speaking louder without mask) or biologically (differences in lung capacity between sexes and ages). Biologically, viral transmission capability could be influenced by less aerosol emission by females and children [37]. These sex-based differences may be also associated with variances in biological pathways such as immune responses against SARS-CoV-2, the expression of X-chromosome–encoded genes [20], or both.

These observations led us to question whether the virus could be transmitted more frequently by men than by women, independently of protection measures, age and socio-economic status. In order to circumvent such differences, we have analyzed the virus transmission in couples who kept neither conjugal distancing during the infection period nor the use of protective measures. Moreover, the mean age did not differ significantly between spouses and both partners had a comparable socio-economic status.

In a previous genetic study with 81 discordant couples for COVID-19, where one person was infected and symptomatic while the partner remained asymptomatic and serum-negative (despite remaining in close contact and sharing the same bed throughout the disease), we observed that there were significantly more women in the asymptomatic group [38]. Subsequently, there were reports of couples where the wife was infected by SARS-CoV-2 and clinically affected while the husband remained asymptomatic despite the close contact throughout the infection period. Some months later, the previously asymptomatic husbands beame infected and symptomatic after contact with male patients.

## Methods

**Fig. 1:**
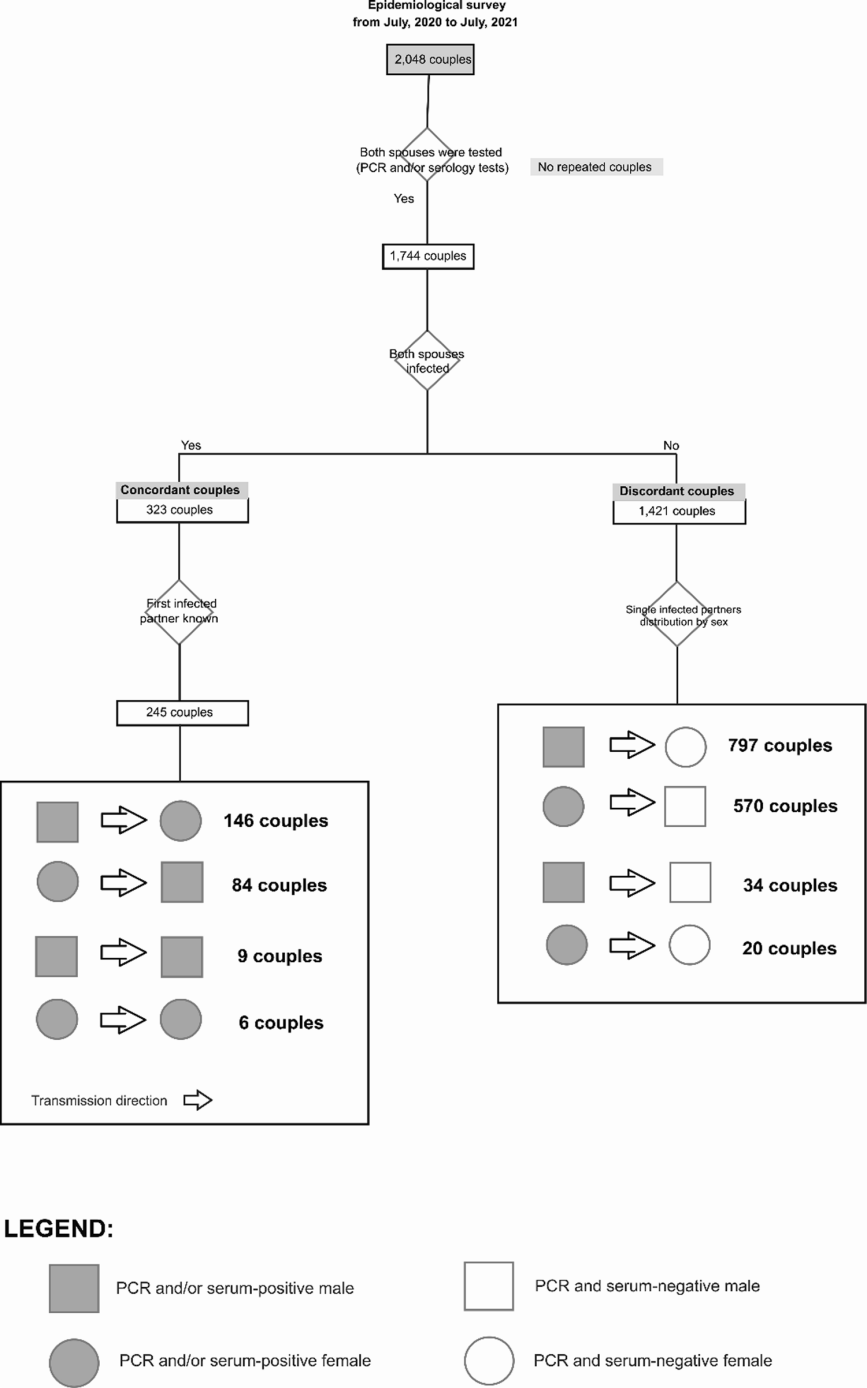
Survey data collection rational diagram

We present an epidemiological survey (from July 2020 to July 2021) including 1744 adult Brazilian couples who were living together during the COVID-19 infection without protective measures. Their ages ranged from 20 to 70 years (overall mean age of 45 years, 44 for women and 46 for men). The positive diagnosis in symptomatic individuals was confirmed by RT-PCR and/or serology for the infected partners while negative results in both tests were confirmed in non-infected partners. These individuals presented complete information related to the infection event, sex, age, and diagnostic tests results. They were divided in two groups: (a) concordant couples where one spouse infected with confirmed COVID-19 transmitted the infection to the partner and (b) discordant couples where one SARS-CoV-2 infected spouse was symptomatic while the partner was not, as confirmed by negative RT-PCR and serology results after viral exposure. The concordant and discordant couples were then subdivided according to which partner was infected first: men or women and women to men. Our whole data collection rationale from partners is presented in Fig. 1.

## Results

As seen in Table 1, concordant couples analysis showed that men are significantly more infectious than women, with an estimated chance of 146/230 = 0.635 (95% confidence interval: 0.569–0.697) of being the first one affected. The corresponding female values were 84/230 = 0.365 (95% confidence interval 0.303–0.431). These differences in male/female values are statistically significant (χ^2^ = 16.713, P $\ll$ 0.0005).

**Table 1:** Numerical description of couple groups composition and chi-square (χ^2^) values

Group	Men	Women	Total	Chi-square (χ^2^) values
Discordant	797	570	1367	37.6950
Concordant	146	84	230	16.7130

The analysis of the group of discordant couples showed again that men are preferentially affected, with a probability estimated as 797/1367 = 0.583 (95% confidence interval: 0.557–0.609). The corresponding female values were 570/1367 = 0.417 (95% confidence interval: 0.391–0.443). The combination of data from concordant and discordant couples showed that the man is preferentially affected in 146 + 797 = 943 occurrences and the woman in 84 + 576 = 660 instances (χ^2^ = 49.962, P $\ll$ 0.0005).

Interestingly, the analysis of COVD-19 reports of the present cohort showed that some 62% among those who mentioned keywords as “fear”, “excessive care”, “pandemic worries” and “anxiety” were sent by females. This observation is consistent with the results of a Brazilian survey with 4693 adults which showed that among 55% who declared that they were worried about the pandemic 62% were females and 45% males [39]. Such observation reporting that men were less worried about the pandemic than women could provide an explanation for their less protective behavior.

## Discussion

All the results obtained in the present study strongly suggest that males are not only more susceptible to COVID-19 severity, as shown in worldwide epidemiological surveys, but they are also more likely to transmit the virus to their partners when compared to females in the household transmission context. The epidemiological findings in the present survey are consistent with the results of other published studies involving couples where one of the partners was infected by their spouses [40, 41]. Female individuals aged between 17 and 65 years were also frequently found to be secondary cases [41].

Aiming to analyze a more homogeneous cohort and since age is an important predictor of severity and risk of death by COVID-19, we focused our survey on couples of comparable ages and economic status and therefore similar access to health care. It is also important to note that the survey was performed before the vaccination was started.

One of the possible current biological hypotheses for such gender variable transmission rate is a differential viral load in saliva, which has been explored as an important clinical measure of disease severity due to its positive association with many COVID-19 inflammatory markers [42]. These factors, together with the higher adoption of hygiene and protective measures among females, may justify the lower transmission rates in this group.

Interestingly, in a recent study of our group [43] it was observed that, although there were no observed gender differences in viral load in nasopharyngeal samples, adult males showed a significantly higher viral load in saliva samples (verified by RT-LAMP viral testing) than adult women.

These observations, together with the evidence of higher aerosol emission by men which makes them more likely to be “superspreaders” than women, support the hypothesis that male individuals are more efficient virus transmitters than females, which is related to biological and behavioral aspects.

This study has some limitations regarding the relatively modest number of couples included in the present cohort when compared with other epidemiological surveys of in-house transmission [40, 41]. Additionally, the couples who responded the questionnaire are on average younger than the mean age of the population since, in Brazil, younger people have more access and familiarity with internet than older adults [44]. Nevertheless, our study brings new knowledge to the field of public health regarding SARS-CoV-2 transmission dynamics.

In short, the present study confirmed the existence of gender differences not only for susceptibility to infection and resistance to COVID-19 but also in the transmission rate.

## Data Availability

Data sharing is not applicable to this article as no datasets were generated during the current study.
